# Neural Mechanisms of Reading Facial Emotions in Young and Older Adults

**DOI:** 10.3389/fpsyg.2012.00223

**Published:** 2012-07-11

**Authors:** Natalie C. Ebner, Marcia K. Johnson, Håkan Fischer

**Affiliations:** ^1^Department of Psychology, University of FloridaGainesville, FL, USA; ^2^Department of Psychology, Yale UniversityNew Haven, CT, USA; ^3^Department of Psychology, Stockholm UniversityStockholm, Sweden; ^4^Aging Research Center, Karolinska InstituteStockholm, Sweden

**Keywords:** emotion, faces, aging, medial prefrontal cortex, amygdala, affective processing, cognitive control

## Abstract

The ability to read and appropriately respond to emotions in others is central for successful social interaction. Young and older adults are better at identifying positive than negative facial expressions and also expressions of young than older faces. Little, however, is known about the neural processes associated with reading different emotions, particularly in faces of different ages, in samples of young and older adults. During fMRI, young and older participants identified expressions in happy, neutral, and angry young and older faces. The results suggest a functional dissociation of ventromedial prefrontal cortex (vmPFC) and dorsomedial prefrontal cortex (dmPFC) in reading facial emotions that is largely comparable in young and older adults: Both age groups showed greater vmPFC activity to happy compared to angry or neutral faces, which was positively correlated with expression identification for happy compared to angry faces. In contrast, both age groups showed greater activity in dmPFC to neutral or angry than happy faces which was negatively correlated with expression identification for neutral compared to happy faces. A similar region of dmPFC showed greater activity for older than young faces, but no brain-behavior correlations. Greater vmPFC activity in the present study may reflect greater affective processing involved in reading happy compared to neutral or angry faces. Greater dmPFC activity may reflect more cognitive control involved in decoding and/or regulating negative emotions associated with neutral or angry than happy, and older than young, faces.

## Introduction

Humans are social-emotional beings. From early on and throughout our life, we are surrounded by social and emotional stimuli that are crucial for our survival and well-being. The ability to correctly interpret other peoples’ feelings, intentions, and behavior, and then respond appropriately and remember such social and emotional information, correctly, such social and emotional information correctly is central for successful social interaction (Baron-Cohen et al., 2000; Grady and Keightley, 2002; Adolphs, 2003). Successful and satisfying social interactions and avoiding social isolation have important consequences for our subjective and objective health and well-being across the entire lifespan (Cornwell and Waite, 2009; Cacioppo et al., 2011). In addition, our interpretation of facial expressions in others has been shown to influence how we attend to, and how well we remember, faces (Ebner and Johnson, 2009; Ebner et al., 2011c).

The effect of aging on reading facial emotions has recently received considerable interest. As summarized in a meta-analysis by Ruffman et al. (2008) that considered data from 962 young (mean age 24 years) and 705 older (mean age 70 years) participants, the predominant pattern was age-related decline in identification of facial emotions (largely comparable findings were also reported for voices, bodies, and matching faces to voices). In particular, compared to young adults, older adults are worse at identifying facial expressions of anger, sadness, and fear. For happiness and surprise, these age-group differences go in the same direction, but are substantially smaller. When interpreting these results, however, one needs to consider that most previous studies have used only one positive expression among various negative expressions. Assuming negative emotions are more difficult to distinguish from each other than from positive emotions, findings of age differences in reading facial emotions may simply reflect older compared to young adults’ greater difficulty in discriminating among more similar negative emotions (Ebner and Johnson, 2009; Ebner et al., 2011c).

In addition, the meta-analysis by Ruffman et al. (2008) suggests that each age group is more accurate in identifying certain expressions than others. In particular, older adults have more difficulty identifying anger, sadness, and fear, compared to disgust, surprise, and happiness, whereas young adults have more difficulty identifying fear and disgust, followed by anger, surprise, sadness, and happiness (Ebner and Johnson, 2009; Murphy and Isaacowitz, 2010; see Isaacowitz et al., 2007, for another meta-analysis).

The literature discusses at least three explanations for age-group differences in reading facial expressions.

(a) *Age-related change in motivational orientation*: According to *Socio-emotional Selectivity Theory* (Carstensen et al., 1999; Carstensen, 2006), due to an increase in perception of future time as limited, older adults become more motivated to maximize positive affect and minimize negative affect in the present, as an adaptive emotion regulation strategy. This is assumed to result in a greater attentional and memory-related focus on, and preference for, positive over negative information (Carstensen and Mikels, 2005; Mather and Carstensen, 2005). This age-related change may be reflected in older adults’ impaired ability to identify negative expressions, whereas the recognition of positive expressions may improve (or at least remain unaffected) with age. This pattern of results is at least partly consistent with the overall literature (see Ruffman et al., 2008). However, findings that older adults are sometimes worse in labeling positive expressions than young adults, and that they are not always worse in recognition of negative expressions (e.g., disgust), are somewhat inconsistent with this theoretical account.
(b) *General age-related cognitive decline*: This account is based on evidence that older adults experience declines in cognition across various functional domains. For instance, normal aging is accompanied by a relative sparing of crystallized abilities (e.g., vocabulary). However, there is broad evidence of age-related declines in processes that involve greater mental effort, self-initiation, inhibitory control, information complexity, novelty, processing speed, and/or working memory (i.e., fluid abilities; Salthouse, 2000; Lustig and Hasher, 2001; Hedden and Gabrieli, 2004). Such age-related changes in general cognitive functioning may have a negative impact on older adults’ ability to identify facial expressions. The little research to date investigating this account does not support this assumption. Rather, the existing studies show that age differences in facial emotion identification remain when accounting for fluid intelligence. For example, age-related reductions in labeling negative expressions were shown to be independent of general age-related cognitive changes in processing speed, basic face processing abilities, and reasoning about non-face stimuli (Sullivan and Ruffman, 2004; Keightley et al., 2006).
(c) *Age-related structural and functional brain changes*: The third account discussed in the literature pertains to evidence that some regions involved in emotional face processing, such as frontal and temporal regions, show substantial structural (Raz and Kennedy, 2009) and functional (Iidaka et al., 2001; Gunning-Dixon et al., 2003; Fischer et al., 2005; Wright et al., 2007) changes with age. These changes may contribute to age-related deficits in the accuracy and speed of reading facial emotions (see Calder et al., 2003; Ruffman et al., 2008). The empirical examination of such effects, however, is still very sparse and the current knowledge about the specific neural processes underlying these effects and potential differences in the neural mechanisms between young and older adults is still very limited.

The particular focus of the present study was on the neural underpinnings of *expression identification of faces* in samples of young and older adults. To our knowledge, only very few fMRI studies to date have explicitly addressed this question as outlined in more detail below (Gunning-Dixon et al., 2003; Williams et al., 2006; Keightley et al., 2007; for a broader discussion of functional neuroimaging evidence on aging and emotion, see St Jacques et al., 2009; Samanez-Larkin and Carstensen, 2011). Importantly, due to design-related issues, none of these previous studies could directly relate young and older adults’ brain activity during facial emotion reading to accuracy or speed of performance. Thus, the present study set out to fill this gap by identifying brain activity in young and older adults during facial emotion reading with happy, neutral, and angry faces, including both young and older faces. Our design allowed us to directly examine the relationship between brain response during task engagement and accuracy and speed of responding in both young and older adults.

Evidence so far suggests involvement of a wide range of neural systems in processing facial emotions, independent of the specific valence, and/or emotion displayed (see Ruffman et al., 2008, for an overview). At the same time, certain brain areas seem to particularly contribute to the processing of individual emotional facial displays and/or seem to be differentially involved in reading positive vs. neutral or negative facial expressions. This suggests that at least partially distinct neural circuits subserve individual emotions and/or different valence of facial expressions.

Ventromedial prefrontal cortex (vmPFC) has been shown to be associated with processing happy faces, possibly in conjunction with amygdala (Keightley et al., 2007; see also Ruffman et al., 2008). This may be due to vmPFC’s function in assessing and representing reward (O’Doherty et al., 2001; Kringelbach and Rolls, 2004). Dorsomedial prefrontal cortex (dmPFC), in contrast, has been shown to be sensitive to various negative expressions (Williams et al., 2006; Keightley et al., 2007). Another area that has been shown to be recruited in emotional face processing is the cingulate cortex (Taylor et al., 1998; Bush et al., 2000; Whalen et al., 2001; Keightley et al., 2003). Both anterior and posterior cingulate cortex are associated with identifying facial expressions of happiness (Salloum et al., 2007), anger (Blair and Cipolotti, 2000), and sadness (Killgore and Yurgelun-Todd, 2004; Salloum et al., 2007).

The majority of neuroimaging studies with young adults have found amygdala activation during viewing of negative faces (and in particular fear and anger but also sadness; Morris et al., 1996; Whalen et al., 2001; Anderson et al., 2003). However, some studies also show increased amygdala activity to positive faces in young adults (Hamann et al., 2002; Pessoa et al., 2002; Winston et al., 2003; Zald, 2003), suggesting that amygdala may have a more general role in directing attention to socially and emotionally relevant cues (Cunningham et al., 2004; Vuilleumier, 2005) than simply and exclusively responding to negative information.

Most neuroimaging studies of processing of positive, neutral, and negative facial expressions conducted so far have limited their investigation to samples of young adults (cf. Gunning-Dixon et al., 2003; Williams et al., 2006; Keightley et al., 2007). Examination of comparable mechanisms between young and older adults as well as differences among the age groups, as both addressed in the present study, will shed more light on the neural and cognitive processes involved in reading facial emotions and their relation to fast and correct facial expression identification. In an investigation of the neural processes involved in facial expression identification in a sample of young and older adults, both structural and functional age-related changes in brain areas associated with this task should be important, as addressed next.

Gradual atrophy is widespread in the brain in aging (Raz et al., 2005; Raz and Kennedy, 2009). At the same time, there is evidence that age-related brain volume reductions and metabolic decline occur earlier and more rapidly in frontal, and particularly in lateral compared to medial frontal, brain regions (Dimberger et al., 2000; Allen et al., 2005; Grieve et al., 2005; Phillips and Henry, 2005). In addition to mPFC, temporal regions such as the amygdala decline less rapidly. Still these areas experience linear volume reductions with age (Mu et al., 1999; Grieve et al., 2005; Wright et al., 2006; Zimmerman et al., 2006).

In addition to age-related structural changes in brain areas associated with processing facial emotions, there also is some evidence of important functional brain changes with age. Consistent evidence of reduced subcortical activity accompanied by increased cortical involvement in older compared to young adults has been shown across various tasks, such as passive viewing of angry and neutral faces (Fischer et al., 2005), gender discrimination of positive, neutral, and negative faces (Iidaka et al., 2001), matching facial emotions of angry and fearful faces (Tessitore et al., 2005), and also age and emotion identification of happy, sad, angry, fearful, disgusted, and neutral faces (Gunning-Dixon et al., 2003; see also Williams et al., 2006; Keightley et al., 2007). This age-related shift toward prefrontal-based and away from amygdala-based facial emotion processing has been interpreted as reflecting more deliberative, controlled processing of emotional information in older than young adults (Satpute and Lieberman, 2006; Williams et al., 2006; see Mather et al., 2004; St Jacques et al., 2010, for similar evidence with scenes and objects) and may reflect age-related increased emotion regulation strategies mediated by frontal brain regions (see St Jacques et al., 2009, for an overview and a discussion).

In particular, using an emotional face viewing task (followed by a facial expression identification task outside the scanner) with blocks of happy and fearful faces in an fMRI study, Williams et al. (2006) found a linear decrease in dmPFC (MNI: *x* = −18, *y* = 22, *z* = 54) activity to happy faces and a linear increase in dmPFC (MNI: *x* = −14, *y* = 36, *z* = 42) activity to fearful faces with increasing age. This finding was interpreted as further support of greater effort and increased controlled processing of negative compared to positive faces with advancing age. Importantly, this shift in mPFC activity for processing positive vs. negative faces was associated with emotional stability: Less dmPFC response to happy faces and more dmPFC response to fearful faces during the face viewing task predicted greater self-reported emotional stability (i.e., lower levels of self-reported neuroticism).

Williams et al. (2006) findings are in line with another study that examined differences between young and older adults’ brain activity in the context of a facial expression identification task and that explicitly differentiated happy from various negative expressions. Keightley and colleagues (Keightley et al., 2007) conducted an event-related fMRI study with faces depicting anger, disgust, fear, happiness, sadness, and surprise. To avoid verbal responses and the high memory load of a multiple-alternative forced-choice response format, participants overtly labeled the faces prior to entering the scanner. They then saw each face again during the scanner task and were asked to silently (re-)label each of them. Largely in line with the literature (Isaacowitz et al., 2007; Ruffman et al., 2008; Ebner and Johnson, 2009), young and older adults performed equally well in identifying happy faces, with ceiling performance in both groups. In addition, young adults outperformed older adults in identifying sadness, anger, and disgust but there were no differences in identifying surprise, fear, or neutral faces.

With respect to the fMRI data, Keightley et al. (2007) reported various findings. One pattern that distinguished happy from other expressions, largely driven by *young adults*, was characterized by greater activity in vmPFC, among other areas (i.e., anterior and posterior cingulate gyrus, left postcentral gyrus, and bilateral middle frontal gyri, bilateral cuneus, precuneus, inferior parietal lobe, and superior temporal gyrus). This was accompanied by decreased activity in left dorsal anterior cingulate gyrus for happy compared to other facial expressions. In addition, at a lower threshold, for young (but not older) adults, there was greater activity in small regions of bilateral amygdala and greater activity in left hippocampus for happy compared to other expressions. A second pattern distinguishing happy from other expressions was largely driven by *older adults*, and was characterized by greater activity in vmPFC among other areas (i.e., lingual gyrus and bilateral premotor cortex; for older adults brain activity in these areas was greater for happy and, to a lesser degree, also disgusted faces when compared with all other expressions). In addition, there was less activity in dorsal anterior cingulate among other areas (i.e., middle and inferior frontal gyrus, somatosensory cortex, middle temporal gyrus, and insula) to happy (and disgust) faces than all other expressions.

Both these brain patterns supported a dorsal/ventral distinction in mPFC that differentiated happy from other facial expressions (note that Keightley et al., 2007, did not differentiate further between the various negative expressions). Importantly, for young and older adults, there was greater activity for happy than other expressions in very similar areas of vmPFC, and, at the same time, greater activity for all other facial expressions compared to happy (and disgust) in very similar regions of dorsal anterior cingulate cortex. Thus, young and older adults partly used different brain networks during (re-)labeling emotional faces. At the same time, however, there was great overlap in the networks recruited by young and older adults, suggesting that the neural processes underlying facial expression identification change little with age. No direct correlational findings between brain activity and accuracy or speed of facial expression reading were reported in the paper.

Taken together, so far most aging studies on processing facial emotions have not explicitly differentiated between different emotions or valences in their analyses (Gunning-Dixon et al., 2003), or have focused exclusively on (different) negative but not positive expressions (Fischer et al., 2005; Tessitore et al., 2005). Moreover, the few studies that have considered both positive and negative faces either did not use facial emotion identification as their orienting task (Iidaka et al., 2001), or conducted facial expression identification outside the scanner (prior to scanning: Keightley et al., 2007; or post scanning: Williams et al., 2006), and thus could not assess correlations between brain activity during task engagement and behavioral performance. There is some evidence in the literature, however, of age differences in attention to, and preference for, positive vs. negative information (Mather and Carstensen, 2005; Isaacowitz et al., 2006; cf. Murphy and Isaacowitz, 2008, for a recent meta-analysis that finds only limited support for a general “positivity effect” in aging). Thus, valence of the expression display is likely to be central for understanding the neural mechanisms involved in facial emotion reading in young and older adults. For example, studies that have used emotional scenes or objects (not faces) have shown greater recruitment of amygdala during the processing of positive than negative scenes in older compared to young adults (Mather et al., 2004; Moriguchi et al., 2011). Also, older compared to young adults were found to recruit vmPFC to a greater extent during processing of positive than negative objects (Leclerc and Kensinger, 2008). And, as reported above, older adults show increased dmPFC activity to negative faces and decreased dmPFC activity to positive faces (Williams et al., 2006). This evidence points to the importance of considering valence as an explicit factor in the design when examining the neural processes involved in reading facial emotions, and when exploring neural-behavioral correlations in samples of young and older adults.

Another important factor, largely ignored in previous studies, is the age of the presented faces. All of the imaging studies on facial emotion reading so far have exclusively used faces of young, and some middle-aged, adults but none has examined the neural mechanisms underlying age differences in reading facial emotions by systematically varying young and older adult faces. However, there is increasing behavioral and neuroimaging evidence of age-of-face effects on processing of faces, such as on attention (e.g., Ebner and Johnson, 2010; Ebner et al., 2011b), evaluation (Ebner et al., 2011a), age estimation (Voelkle et al., 2012), and memory (see Rhodes and Anastasi, 2012, for a meta-analysis; see also Ebner and Johnson, 2009; He et al., 2011). In particular, recent behavioral studies that examined the impact of the age of the face on young and older adults’ ability to correctly identify facial emotions suggest that performance in both age groups is better for young than older faces (Ebner and Johnson, 2009; Ebner et al., 2011c; Riediger et al., 2011). One possibility is that expressions in young compared to older faces are easier to read because emotion cues are more explicit and less ambiguous in young than (more wrinkled and thus more complex) older faces (see Ebner and Johnson, 2009; Ebner et al., 2011b).

The present study had the following two major aims (see Table 1 for a summary): *Research Aim 1* was to examine brain activity in vmPFC, dmPFC, and amgydala during facial expression identification as a function of facial expression and age of face, respectively, across young and older adults. As outlined above, previous neuroimaging evidence suggests a role of vmPFC and dmPFC in facial expression reading in young and older adults and amygdala involvement in young adults (Keightley et al., 2007). Moreover, behavioral studies suggest that happy and young faces are easier to read than angry (or neutral) and older faces for young and also older adults (Ebner and Johnson, 2009; Ebner et al., 2011c). Based on this previous evidence, *Hypothesis 1a* predicted greater activity in vmPFC to happy than angry (or neutral) faces, and similarly to young than older faces, for both young and older adults. Even though various studies suggest amygdala activation during viewing of negative faces (Whalen et al., 2001), Keightley et al. found greater amygdala activation, at least in young adults, to happy than various other (negative) facial expressions in a facial expression identification task quite similar to the one used in the present study. Thus, *Hypothesis 1b* predicted greater amygdala activity to happy than angry (or neutral) faces, and also to young than older faces, for both young and older adults. *Hypothesis 1c* predicted greater dmPFC activity to angry (or neutral) than happy faces, and to older than young faces, across both young and older adults. Based on previous literature, reviewed above, suggesting some age-group differences in vmPFC, dmPFC, and amygdala activity during facial expression reading (Gunning-Dixon et al., 2003; Williams et al., 2006; Keightley et al., 2007), *Hypothesis 1d* predicted greater dmPFC activity to angry (or neutral) than happy faces in older than young participants. This age difference may be due to increased controlled processing of negative relative to positive information with age (Williams et al., 2006) and/or older adults’ particular difficulty decoding anger from faces (Ruffman et al., 2008; see also Ebner and Johnson, 2009; Ebner et al., 2011c).

**Table 1:** **Overview of the central research aims and study predictions**.

Research aim	Specific study prediction	Previous evidence
*Research Aim 1*: Brain activity in vmPFC, dmPFC, and amgydala during facial expression identification as a function of facial expression and age of face in young and older adults	*Hypothesis 1a*: Greater vmPFC activity to happy than angry (or neutral) faces and to young than older faces across age groups	e.g., Gunning-Dixon et al. (2003), Williams et al. (2006), Keightley et al. (2007), Ruffman et al. (2008), Ebner and Johnson (2009), Ebner et al. (2011c)
	*Hypothesis 1b*: Greater amygdala activity to happy than angry (or neutral) faces and to young than older faces across age groups	
	*Hypothesis 1c*: Greater dmPFC activity to angry (or neutral) than happy faces and to older than young faces across age groups	
	*Hypothesis 1d*: Greater dmPFC activity to angry (or neutral) than happy faces in older than young adults
*Research Aim 2*: Brain-behavior correlations in vmPFC, dmPFC, and amygdala for different facial expressions and different age of faces in young and older adults	*Hypothesis 2a*: Positive correlations between vmPFC activity to happy relative to angry (or neutral) faces and ability of identifying happy relative to angry (or neutral) faces in young and older adults; similar pattern predicted for young relative to older faces	e.g., Williams et al. (2006), Ruffman et al. (2008), Ebner et al. (2011c)
	*Hypothesis 2b*: Positive correlations between amygdala activity to happy relative to angry (or neutral) faces and ability of identifying happy relative to angry (or neutral) faces in young and older adults; similar pattern predicted for young relative to older faces	
	*Hypothesis 2c*: Negative correlations between dmPFC activity to angry (or neutral) relative to happy faces and ability of identifying angry (or neutral) vs. happy faces in young and older adults; similar pattern predicted for older relative to young faces	

The expected ventral/dorsal distinction in mPFC (see *Hypotheses 1a* and *1c*) may reflect greater “ease” of (i.e., less controlled) processing of happy than angry (or neutral) faces and young than older faces (see Williams et al., 2006). Consequently, *Research Aim 2* was to examine the brain-behavior correlations in vmPFC, dmPFC, and amygdala for the facial expressions in relation to each other as well as young vs. older faces in samples of young and older adults. In particular, *Hypothesis 2a* predicted a positive correlation between vmPFC activity to happy relative to angry (or neutral) faces and accuracy, as well as speed, of identifying happy relative to angry (or neutral) expressions in both young and older adults. A similar pattern was predicted for young compared to older faces. In addition, comparable correlations were expected for amygdala activity (*Hypothesis 2b*). *Hypothesis 2c*, in contrast, predicted a negative correlation between dmPFC activity to angry (or neutral) relative to happy faces and accuracy, as well as speed, of identifying angry (or neutral) relative to happy expressions in both young and older participants. Again, a comparable pattern was predicted for older compared to young faces.

The focus of the present paper on mPFC and amygdala as regions of interest (ROI) was motivated by evidence outlined above that these areas appear to be particularly involved in facial emotion reading in young and older adults (Keightley et al., 2007). In addition, these regions have been shown to be involved in thinking about the self in both young and older adults (Gutchess et al., 2007; Mitchell et al., 2009; Ebner et al., 2011a). That is, areas of mPFC are recruited when young (Amodio and Frith, 2006; Mitchell, 2009; Van Overwalle, 2009) and older (Gutchess et al., 2007; Ebner et al., 2011a) adults “mentalize” about their own or other people’s intentions, thoughts, feelings, and preferences, or empathize with them (Völlm et al., 2006), which are processes that appear particularly relevant when attempting to decode other people’s emotions and feelings from facial displays as in the present study. In addition, these brain regions show only moderate age-related structural changes (Raz and Kennedy, 2009) and show largely intact functional patterns in older adults (Gutchess et al., 2007; Wright et al., 2008; Ebner et al., 2011a, in preparation), even in studies that find overall lower activity in these regions in older than young adults (Mather et al., 2004; Mitchell et al., 2009). Also, as discussed above, there is evidence of an age-related shift from amygdala to more frontal regions with aging during processing of facial emotions (Iidaka et al., 2001; Gunning-Dixon et al., 2003; Fischer et al., 2005; St Jacques et al., 2009). This evidence combined makes mPFC and amygdala particularly interesting candidates in an examination of the neural mechanisms underlying facial emotion reading in samples of young and older adults.

## Materials and Methods

### Participants

Participants were healthy young adults [*n* = 30 (16 females), *M* = 25.1 years (SD = 3.4; range = 20–31)] and healthy, active, independently living older adults [*n* = 32 (18 females), *M* age = 68.2 years (SD = 2.5; range = 65–74)]. Due to technical problems with the response pad, behavioral data for the task were lost for one older woman and one older man. Thus, all behavioral data were based on *N* = 60 participants. Young [*M* = 14.8 years (SD = 2.1; range = 12–19)] and older [*M* = 14.5 years (SD = 3.7; range = 9–27)] participants did not differ in their years of education [*F*(1,59) = 0.21, *p* = 0.652, ${\text{η}}_{p}^{2}$ = 0.00]. Table 2 presents descriptive information and age-group differences in cognitive and affective measures for both age groups. There were no differences on MMSE scores, verbal fluency, depression, or anxiety. However, young participants scored better than older participants in processing speed, episodic memory, and working memory, and older participants scored better in vocabulary than young participants. Participants were all in good health, with no known history of stroke, heart disease, or primary degenerative neurological disorder, and were right-handed native Swedish speakers. They all had normal or corrected-to-normal vision (using MR-compatible eyeglasses) and none were known to take psychotropic medications. A radiologist screened both a T1-weighted and T2-weighted structural image of the older participants to rule out gray and white matter lesions and/or abnormal amount of atrophy.

**Table 2:** **Means (*M*) and standard deviations (SD) and age-group differences for cognitive and affective measures**.

Measures	Young participants *M* (SD)	Older participants *M* (SD)	Age-group differences
**COGNITIVE FUNCTIONING**			
MMSE	29.3 (0.69)	28.9 (0.91)	*F*(1, 59) = 3.08, *p* = 0.084, ${\text{η}}_{p}^{2}$ = 0.05
LCT	11.0 (2.06)	8.44 (2.01)	*F*(1, 59) = 25.6, *p* < 0.001, ${\text{η}}_{p}^{2}$ = 0.29
FWRT	10.0 (2.34)	7.16 (1.85)	*F*(1, 59) = 28.4, *p* < 0.001, ${\text{η}}_{p}^{2}$ = 0.33
2-Back	8.44 (1.38)	6.27 (1.95)	*F*(1, 57) = 24.2, *p* < 0.001, ${\text{η}}_{p}^{2}$ = 0.30
SST	22.6 (3.68)	26.1 (2.53)	*F*(1, 59) = 19.5, *p* < 0.001, ${\text{η}}_{p}^{2}$ = 0.25
VF	15.1 (4.97)	16.5 (6.95)	*F*(1, 59) = 0.9, *p* = 0.348, ${\text{η}}_{p}^{2}$ = 0.02
**AFFECTIVE FUNCTIONING**			
GDS	1.37 (1.63)	1.45 (2.51)	*F*(1, 59) = 0.02, *p* = 0.877, ${\text{η}}_{p}^{2}$ = 0.02
STAI	30.5 (5.35)	28.3 (6.61)	*F*(1, 58) = 1.98, *p* = 0.165, ${\text{η}}_{p}^{2}$ = 0.03

*MMSE, Mini Mental State Examination; dementia screening; maximum possible = 30 (higher score representing better cognitive performance); Folstein et al. (1975). LCT, Letter Comparison Task; processing speed; mean number of correct comparisons of two letter strings within 30 s; Salthouse and Babcock (1991). FWRT, Free Word Recall Task; episodic memory; recall of a list of 16 words (e.g., envelope, guitar) after 120 s; newly developed. 2-Back, 2-Back Digits Task; working memory; mean number of correct responses; maximum possible = 10; Kirchner (1958). SST, Swedish Synonym Task; underlining of one synonym to target word out of four choices; maximum possible = 30; Dureman (1960). VF, Verbal Fluency Task; verbal fluency; mean number of correctly generated words starting with a given letter (A and F, respectively), within 60 s; Lezak (1995). GDS, Geriatric Depression Scale; depression screening; maximum possible = 20 (higher scores represent more depressive symptoms); Brink et al. (1982); Gottfries (1997; Swedish version). STAI, State-Trait Anxiety Inventory; state and trait anxiety; maximum possible = 80 (higher scores representing greater state-trait anxiety); Spielberger et al. (1983). There were missing data for questionnaires and covariate measures for one older woman; missing data for 2-Back for one young man and one older woman; missing data for STAI for one young man*.

### Stimuli

Stimuli were taken from the FACES database (for detailed information, see Ebner et al., 2010). Face stimuli were digital, high-quality, color, front-view head shots on gray background, all standardized in terms of production and general selection procedure. Each participant saw 32 happy, 32 neutral, and 32 angry faces, each a unique identity, with equal numbers of young (18–31 years) and older (69–80 years) male and female faces. Stimulus presentation and response collection (accuracy and response time) were controlled using E-Prime (Schneider et al., 2002).

### Procedure, measures, and design

The ethics committee at the Karolinska Institute approved the protocol; informed consent was obtained from all participants at the beginning of the study session. The data reported here were embedded in a larger project. Only a subset of variables is reported in this paper. In the first session, approximately one week before scanning, participants filled in several paper-and-pencil questionnaires (i.e., Demographic Questionnaire, MMSE, GDS, STAI) and worked on various computer tasks (i.e., LCT, FWRT, 2-Back, SST, VF; see Table 2).

**Figure 1:**
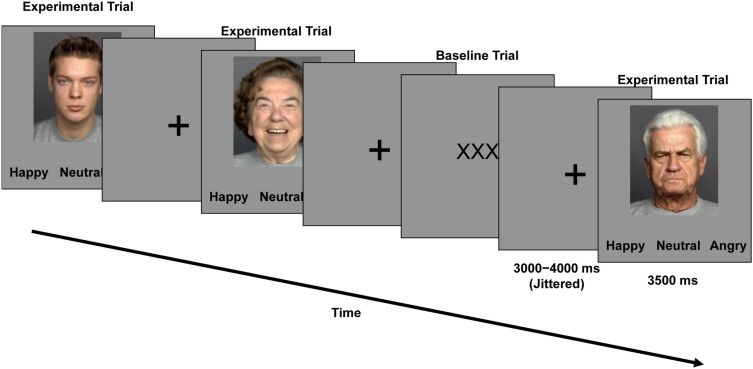
**Trial event timing and sample faces used in the *Facial Expression Identification Task***.

During the second session (fMRI), participants worked on the *Facial Expression Identification Task* (Figure 1). This task had a mixed 2 (*age of participant*: young, older) × 3 (*facial expression*: happy, neutral, angry) × 2 (*age of face*: young, older) factorial design, with *age of participant* as a between-subjects factor and *facial expression* and *age of face* as within-subjects factors. As shown in Figure 1, participants saw faces, one at a time. Each face was presented for 3500 ms. Participants were asked to indicate whether the displayed face showed a happy, neutral, or angry expression by pressing one of three response buttons on a button box (index finger for “happy,” middle finger for “neutral,” and ring finger for “angry” expressions) as fast and accurately as possible. Response options appeared in black on a gray background below the faces and were always presented in the same order. In between faces, a black fixation cross appeared on a gray background on the screen. The inter-stimulus interval (ISI) pseudo-randomly varied between 3000 and 4000 ms in 250 ms increments (mean ISI = 3500 ms). In one-third of the trials (48 out of a total of 144 trials), “low-level baseline events” of three black Xs on a gray background were presented. Participants pressed any one of the three buttons that they also used for labeling the facial expressions to indicate appearance of a low-level baseline trial.

The presentation order of face identities was identical for each participant with facial expressions counterbalanced across participants (each participant only saw each face with one expression). Lists were pseudo-randomized with the constraints that no more than two faces of the same category (i.e., age, gender, facial expression) were repeated in a row. The presentation order of faces and low-level baseline events was pseudo-randomized with the constraint that no more than three faces and no more than two baseline trials were presented in a row. The task started with four practice trials. It was split into two runs, each of them lasting for 8.4 min. At the end of the session, participants were debriefed and financially compensated for participation.

### Imaging details

Images were acquired using a 3T Siemens Magnetom TrioTim scanner at Huddinge Hospital, Stockholm, Sweden. After localizer scans, two runs of 160 functional images each were acquired with a T2*-weighted echo-planar sequence (ep2d_bold; TR = 2500 ms, TE = 40 ms, flip angle = 90°, FoV = 230 mm, voxel size = 3 mm × 3 mm × 3 mm). Thirty-nine oblique axial slices were positioned parallel to the AC-PC line, and acquired interleaved. A 1 mm × 1 mm × 1 mm T1-weighted image was used for co-registration with functional images (MP-RAGE; TR = 1900 ms, TE = 2.52 ms, FoV = 256 mm).

### fMRI analyses

Data from this event-related fMRI study was analyzed using Statistical Parametric Mapping (SPM5; Wellcome Department of Imaging Neuroscience). Pre-processing included slice timing correction, motion correction, co-registration of functional images to the participant’s anatomical scan, spatial normalization, and smoothing [9 mm full-width half maximum (FWHM) Gaussian kernel]. Spatial normalization used a study-specific template brain composed of the average of the young and older participants’ T1 structural images (detailed procedure for creating this template is available from the authors). Functional images were re-sampled to 3 mm isotropic voxels at the normalization stage, resulting in image dimensions of 53 × 63 × 46.

For the fMRI analysis, first-level, single-subject statistics were modeled by convolving each trial with the SPM canonical hemodynamic response function to create a regressor for each condition (young happy, young neutral, young angry, older happy, older neutral, older angry). Parameter estimates (beta images) of activity for each condition and each participant were then entered into a second-level random-effects analysis using a mixed 2 (*age of participant*) × 3 (*facial expression*) × 2 (*age of face*) ANOVA, with *age of participant* as a between-subjects factor and *facial expression* and *age of face* as within-subjects factors. From within this model, the following six *T*-contrasts were specified across the whole sample to address *Hypotheses 1a*–*1c* (see Table 1): (a) *happy faces* > *neutral faces*, (b) *happy faces* > *angry faces*, (c) *neutral faces* > *happy faces*, (d) *angry faces* > *happy faces*, (e) *young faces* > *older faces*, (f) *older faces* > *young faces*. In addition, the following two *F*-contrasts examining interactions with *age of participant* were conducted to address *Hypothesis 1d* (see Table 1): (g) *happy faces* vs. *neutral faces by age of participant*, (h) *happy faces* vs. *angry faces* *by age of participant*. Analyses were based on all trials, not only on those with accurate performance. Young and older participants’ accuracy of reading the facial expressions was quite high for all conditions (ranging between 98.5 and 88.5%; see Table 3); that is, only few errors were made. Nevertheless, consideration of all, and not only correct, trials in the analyses leaves the possibility that for some of the facial expressions the subjective categorization may have differed from the objectively assigned one (see Ebner and Johnson, 2009, for a discussion).

**Table 3:** **Means (*M*) and standard deviations (SD) for accuracy (%) and response time (ms) in expression identification of happy, neutral, and angry young and older faces for young and older participants**.

	Accuracy (%) *M* (SD)		Response time (ms) *M* (SD)	
	Young participants	Older participants	Young participants	Older participants
**HAPPY FACES**				
Young faces	95.8 (9.5)	98.5 (3.6)	1077 (145)	1234 (241)
Older faces	94.6 (10.6)	96.7 (5.9)	1159 (216)	1250 (212)
**NEUTRAL FACES**				
Young faces	92.1 (11.9)	96.0 (6.0)	1292 (241)	1318 (228)
Older faces	90.2 (10.7)	91.3 (9.9)	1469 (295)	1567 (267)
**ANGRY FACES**				
Young faces	94.8 (9.6)	94.6 (10.2)	1340 (251)	1527 (288)
Older faces	88.5 (12.9)	91.9 (9.9)	1451 (295)	1638 (293)

We conducted four sets of analyses on selected *a priori* ROIs defined by the WFU PickAtlas v2.4 (Maldjian et al., 2003, 2004,^1^; based on the Talairach Daemon) and using different thresholds: (1) For all *T*-contrasts listed above, we used a mPFC ROI mask that comprised bilateral medial frontal gyrus and anterior cingulate gyrus based on the anatomic labels specified in the WFU PickAtlas. For this set of analyses we used a threshold of 10 contiguous voxels each significant at *p* < 0.001, uncorrected for multiple comparisons. (2) All *T*-contrasts were also examined using an amygdala ROI mask comprising bilateral amygdala as specified in the WFU PickAtlas. For examination of this circumscribed, small ROI, we used a threshold of *p* < 0.05, uncorrected, with the number of contiguous voxels unspecified. (3) For all *F*-contrasts (i.e., interactions with participant age), we used the mPFC ROI at a threshold of 10 contiguous voxels, each significant at *p* < 0.05, uncorrected. This lowered threshold was used to increase the sensitivity to detect significant interaction effects with *age of participant*. (4) Finally, for all *F*-contrasts, assessing interactions with participant age, we also conducted analyses using the amygdala ROI, again at a lowered threshold (*p* < 0.05, uncorrected, number of contiguous voxels unspecified).

For each region of activation identified by a contrast, beta values were extracted for each participant to produce a single value for each condition of interest. These values are depicted in the bar graphs and the scatter plots of Figures 3–5. In the fashion of follow-up *F*- and *t*-tests in analysis of variance (ANOVA), subsequent statistical comparisons of these values (*p* < 0.05) were conducted using IBM SPSS Statistics Version 20 to aid interpretation of the activations. Montreal Neurological Institute (MNI) coordinates are reported. Anatomical localization were verified using the Talairach Daemon (Lancaster et al., 1997, 2000) on coordinates transformed using *icbm2tal*,^2^ and labels were confirmed visually using the Talairach and Tournoux (1988) atlas.

## Results

### Behavioral data

**Figure 2:**
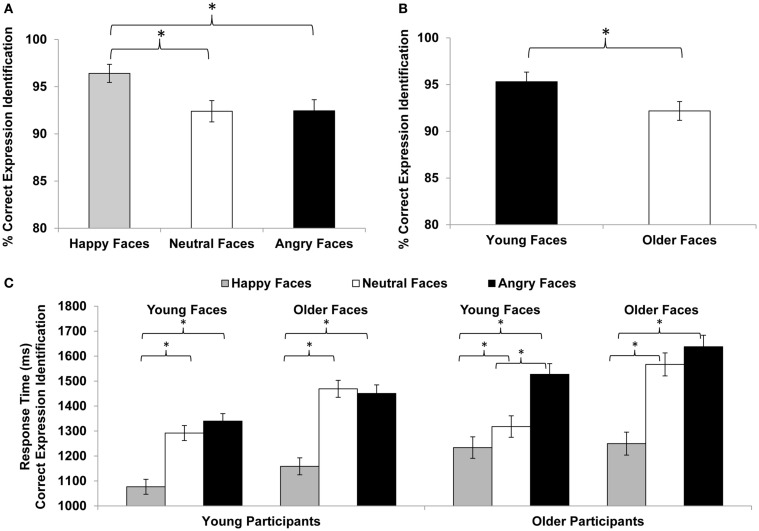
**Facial expression identification (% correct) for (A) happy, neutral, vs. angry faces and (B) young vs. older faces**. **(C)** Response time (ms) for facial expression identification in young and older participants for happy, neutral, and angry young and older faces. Error bars represent standard errors of condition mean differences; *p ≤ 0.05.

Compliance with the task in the scanner was high, with a button press on 97% of the faces trials (76% of low-level baseline trials). In a first step, we conducted separate mixed 2 (*age of participant*: young, older) × 3 (*facial expression*: happy, neutral, angry) × (*age of face*: young, older) repeated-measures ANOVAs on accuracy and response time of accurate responses, respectively (see Table 3; Figure 2). For accuracy, neither of the three- or two-way interactions was significant. The only significant effects were the main effects for *facial expression* [*F*(2,57) = 16.56, *p* < 0.001, ${\text{η}}_{p}^{2}$ = 0.37; Figure 2A] and *age of face* [*F*(1,58) = 23.10, *p* < 0.001, ${\text{η}}_{p}^{2}$ = 0.29; Figure 2B]. Overall, participants were very good at identifying the facial expressions. Moreover, they were better at reading happy (*M* = 96.4%, SD = 7.5) than neutral (*M* = 92.4%, SD = 8.8) or angry (*M* = 92.4%, SD = 9.3) expressions and were better at reading young (*M* = 95.3%, SD = 8.0) than older (*M* = 92.2%, SD = 7.8) faces.

For speed of responding (see Table 3 and Figure 2C), the main effects for *facial expression* [*F*(2,57) = 98.56, *p* < 0.001, ${\text{η}}_{p}^{2}$ = 0.78], *age of face* [*F*(1,58) = 103.66, *p* < 0.001, ${\text{η}}_{p}^{2}$ = 0.64], and *age of participant* [*F*(1,58) = 5.12, *p* = 0.027, ${\text{η}}_{p}^{2}$ = 0.08] were significant. There also were significant interactions for *facial expression* × *age of face* [*F*(2,57) = 17.94, *p* < 0.001, ${\text{η}}_{p}^{2}$ = 0.39], *facial expression* × *age of participant* [*F*(2,57) = 3.21, *p* < 0.048, ${\text{η}}_{p}^{2}$ = 0.10], and *facial expression* × *age of face* × *age of participant* [*F*(2,57) = 3.12, *p* < 0.052, ${\text{η}}_{p}^{2}$ = 0.10]. Although young and older participants did not show a behavioral performance difference with respect to accuracy, older participants (*M* = 1422 ms, SD = 208) were overall slower to respond than young participants (*M* = 1298 ms, SD = 218). In particular, older compared to young participants were slower in responding to happy (young participants: *M* = 1118 ms, SD = 166; older participants: *M* = 1242 ms, SD = 219) and angry (young participants: *M* = 1395 ms, SD = 263; older participants: *M* = 1582 ms, SD = 276) but not neutral (young participants: *M* = 1381 ms, SD = 254; older participants: *M* = 1442 ms, SD = 230) faces. In line with the accuracy data, response time to young faces (*M* = 1298 ms, SD = 212) was faster than response time to older faces (*M* = 1422 ms, SD = 237). And, collapsed across young and older adults, response time to happy faces (*M* = 1180 ms, SD = 203) was faster than response time to neutral faces (*M* = 1411 ms, SD = 242), which was faster than response time to angry faces (*M* = 1489 ms, SD = 283). However, the significant difference between neutral and angry faces held only for older [*t*(29) = −3.29, *p* = 0.003] but not young [*t*(29) = −0.61, *p* = 0.550] participants and was driven by a faster responses to young neutral than young angry faces [*t*(29) = −4.55, *p* < 0.001]; the difference between older neutral and older angry faces was not significant [*t*(29) = −1.38, ns; see Figure 2C].

### fMRI data

The results section is structured along the two central aims of the study (see Table 1). We start by reporting results pertaining to brain activity in vmPFC, dmPFC, and amgydala during facial expression identification as a function of the facial expression and the age of the face, respectively, across the whole sample (*Research Aim 1*). This is followed by an examination of the correlations between brain response in vmPFC, dmPFC, and amygdala and behavioral performance in the facial expression identification task for the different facial expressions and different age of faces, respectively, in both young and older participants (*Research Aim 2*).

#### Brain activity in vmPFC, dmPFC, and amygdala

##### Happy faces > neutral faces and happy faces > angry faces and young faces > older faces across the whole sample

**Figure 3:**
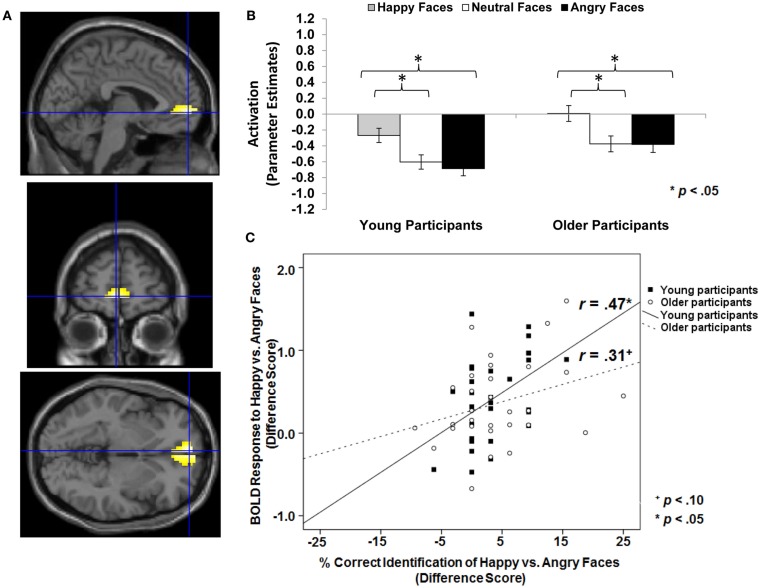
**Area of vmPFC where happy faces > angry faces (*T*-contrast): (A) Left ventral medial frontal gyrus, anterior cingulate (BA 10; MNI: *x* = −3, *y* = 57, *z* = −3; cluster size: 49 voxels; maximum *T*-value for cluster: 5.11)**. The region of activation represents the *T*-map of the contrast; it is displayed on the standard reference brain in SPM. The crosshair indicates the peak voxel (local maximum) within the region of activation. **(B)** Bar graphs show the mean left vmPFC parameter estimates (beta values) separately for facial expression and age of participant (across age of face); betas for this region of activation identified by the *T*-contrast *happy faces* > *angry faces* were extracted for each individual from a 5-mm sphere around the local maximum within the region of activation and averaged to produce a single value for each condition of interest, respectively. **(C)** Mean difference in participants’ left vmPFC BOLD response to happy relative to angry faces in relation to the percentage of correctly identified happy relative to angry faces for young and older participants, respectively.

As a first step, we were interested in testing whether vmPFC activity was greater to happy than neutral or angry faces across the whole sample (see Table 1; *Hypothesis 1a*). As presented in Table 4 (section A, Analysis across whole sample), similar areas of bilateral *vmPFC* showed greater BOLD response to happy compared to neutral (MNI: *x* = −3, *y* = 63, *z* = 0) and happy compared to angry (MNI: *x* = −3, *y* = 57, *z* = −3) faces. Figure 3A shows brain activity in left vmPFC (MNI: *x* = −3, *y* = 57, *z* = −3) for the contrast *happy faces* > *angry faces*. Follow-up paired-sample *t*-tests collapsed across the whole sample on extracted beta values at the peak voxel of activation showed that left vmPFC activity was greater for happy than angry [*t*(61) = 6.32, *p* < 0.001] and neutral [*t*(61) = 5.01, *p* < 0.001] faces. Figure 3B presents these extracted beta values separately for young and older adults. The pattern of results was quite comparable for the two age groups with both young [*t*(29) = 4.45, *p* < 0.001] and older [*t*(31) = 4.42, *p* < 0.001] participants showing greater left vmPFC activity for happy than angry faces. Note that vmPFC deactivation is often seen during cognitive tasks and vmPFC activation during rest (Raichle et al., 2001). Self-relevant and/or emotional processing has been associated with activation in vmPFC (Johnson et al., 2006) or with less deactivation (Ames et al., 2008), as observed in the present study.

**Table 4:** **Results of ROI analyses: activity in mPFC and amygdala during facial expression identification to happy relative to neutral or angry and young relative to older faces (across whole sample and in interaction with participant age)**.

Hemi	BA	Anatomical area	Activation peak			*T*-value/*F*-value	# Vox
			*x*	*y*	*z*	
**(A) Analysis across whole sample**							
*Happy faces > neutral faces across whole sample*							
B	10	Medial frontal gyrus	−3	63	0	5.68	164
R		Amygdala	24	−9	−12	2.75	6
*Happy faces > angry faces across whole sample*							
**B**	**10**	**Medial frontal gyrus, anterior cingulate**	−**3**	**57**	−**3**	**5.11**	**49**
R		Amygdala	24	−9	−18	1.87	3
*Young faces > older faces across whole sample*							
**–**							
*Neutral faces > happy faces across whole sample*							
**B**	**8, 6**	**Superior frontal gyrus, medial frontal gyrus**	−**6**	**24**	**48**	**5.33**	**69**
*Angry faces > happy faces across whole sample*							
L	6	Superior frontal gyrus, medial frontal gyrus	−6	15	51	6.46	312
*Older faces > young faces across whole sample*							
**B**	**8, 32**	**Medial frontal gyrus, anterior cingulate gyrus, superior frontal gyrus**	−**3**	**33**	**39**	**4.94**	**102**
**(B) Interaction with participant age**							
*Happy faces vs. neutral faces by participant age*							
R	24	Cingulate gyrus	6	9	27	8.32	11
*Happy faces vs. angry faces by participant age*							
**R**	**24**	**Anterior cingulate, medial frontal gyrus**	**12**	**39**	−**3**	**7.67**	**52**
**B**	**8, 6**	**Medial frontal gyrus, superior frontal gyrus**	−**6**	**27**	**51**	**9.50**	**23**
R	6	Medial frontal gyrus	15	3	54	8.45	19

**Figure 4:**
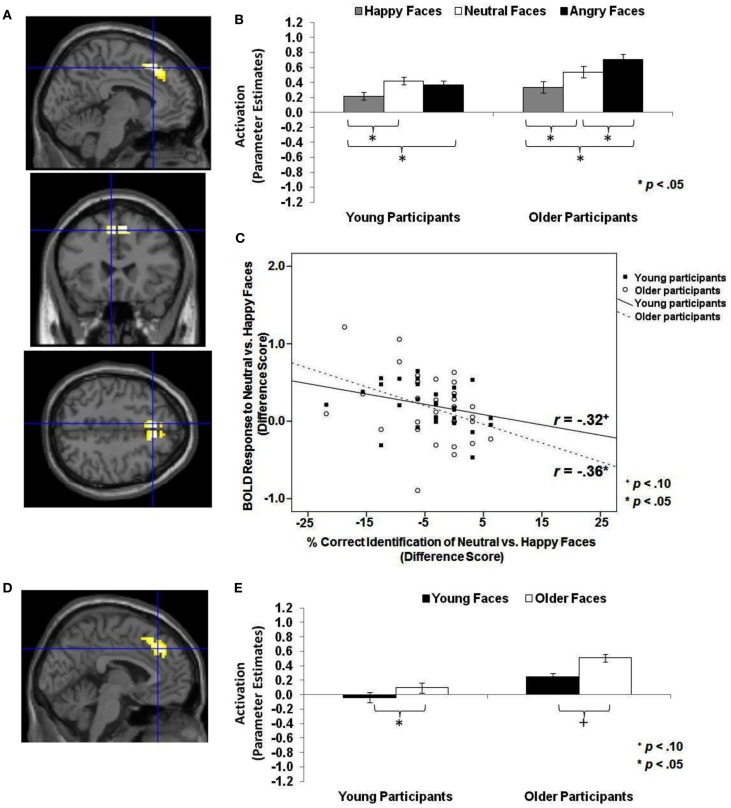
**Area of dmPFC where neutral faces > happy faces and older faces > young faces (*T*-contrasts): neutral faces > happy faces: (A) Left superior frontal gyrus, medial frontal gyrus (BA 8, 6; MNI: *x* = −6, *y* = 24, *z* = 48; cluster size: 69 voxels; maximum *T*-value for cluster: 5.33)**. The region of activation represents the *T*-map of the contrast; it is displayed on the standard reference brain in SPM. The crosshair indicates the peak voxel (local maximum) within the region of activation. **(B)** Bar graphs show the mean left dmPFC parameter estimates (beta values) separately for facial expression and age of participant (across age of face); betas for this region of activation identified by the *T*-contrast *neutral faces* > *happy faces* were extracted for each individual from a 5-mm sphere around the local maximum within the region of activation and averaged to produce a single value for each condition of interest, respectively. **(C)** Mean difference in participants’ left dmPFC BOLD response to neutral relative to happy faces in relation to the percentage of correctly identified neutral relative to happy faces for young and older participants, respectively. *older faces* > *young faces*: **(D)** Left medial frontal gyrus, anterior cingulate, superior frontal gyrus (BA 8, 32; MNI: *x* = −3, *y* = 33, *z* = 39; cluster size: 102 voxels; maximum *T*-value for cluster: 4.94). The region of activation represents the *T*-map of the contrast; it is displayed on the standard reference brain in SPM. The crosshair indicates the peak voxel (local maximum) within the region of activation. **(E)** Bar graphs show the mean left dmPFC parameter estimates (beta values) separately for age of face and age of participant (across facial expression); betas for this region of activation identified by the *T*-contrast *older faces* > *young faces* were extracted for each individual from a 5-mm sphere around the local maximum within the region of activation and averaged to produce a single value for each condition of interest, respectively.

**Figure 5:**
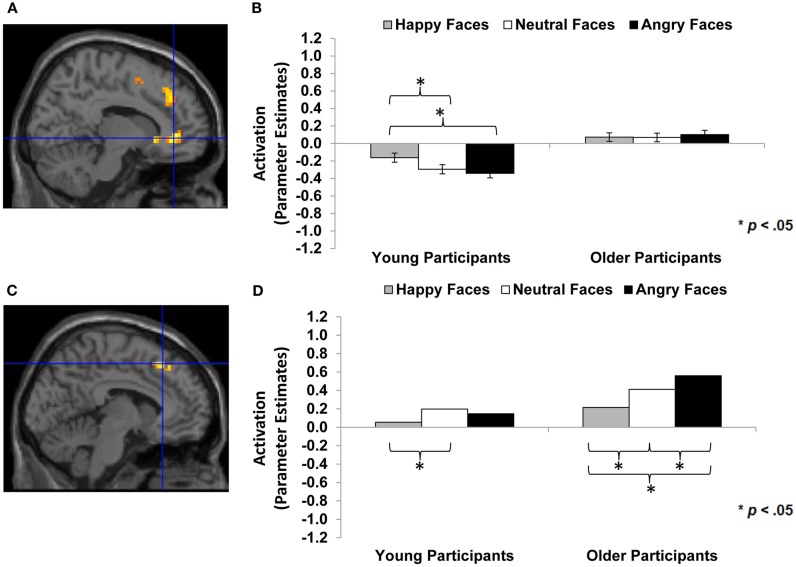
**Area of vmPFC and dmPFC showing happy vs. angry faces by age of participant interaction (*F*-contrast): (A) Right medial frontal gyrus, anterior cingulate (BA 24; MNI: *x* = 12, *y* = 39, *z* = −3; cluster size: 52 voxels; maximum *F*-value for cluster: 7.67)**. The region of activation represents the *F*-map of the contrast; it is displayed on the standard reference brain in SPM. The crosshair indicates the peak voxel (local maximum) within the region of activation. **(B)** Bar graphs show the mean right vmPFC parameter estimates (beta values) separately for facial expression and age of participant (across age of face); betas for this region of activation identified by the *F*-contrast *happy* vs. *angry faces by age of participant* were extracted for each individual from a 5-mm sphere around the local maximum within the region of activation and averaged to produce a single value for each condition of interest, respectively. **(C)** Left medial frontal gyrus, superior frontal gyrus (BA 8, 6; MNI: *x* = −6, *y* = 27, *z* = 51; cluster size: 23 voxels; maximum *F*-value for cluster: 9.50). The region of activation represents the *F*-map of the contrast; it is displayed on the standard reference brain in SPM. The crosshair indicates the peak voxel (local maximum) within the region of activation. **(D)** Bar graphs show the mean left dmPFC parameter estimates (beta values) separately for facial expression and age of participant (across age of face); betas for this region of activation identified by the *F*-contrast *happy* vs. *angry faces by age of participant* were extracted for each individual from a 5-mm sphere around the local maximum within the region of activation and averaged to produce a single value for each condition of interest, respectively.

*Analyses across whole sample (*T*-contrasts) used a threshold of 10 contiguous voxels, each at *p* < 0.001, uncorrected, for mPFC and *p* < 0.05, uncorrected, with number of contiguous voxels unspecified for amygdala. Age of participant interaction analyses (*F*-contrasts) used a threshold of 10 contiguous voxels, each at *p* < 0.05, uncorrected, for mPFC and *p* < 0.05, uncorrected, with number of contiguous voxels unspecified for amygdala. Areas printed in **bold** are shown in Figures 3–5, respectively. MNI coordinates (*x*, *y*, *z*) and maximum *T*-value/*F*-value are given for the peak voxel (local maximum) within each region of activation. Hemi, hemisphere; L, left; R, right; B, bilateral; BA, Brodmann area; # vox, number of voxels in cluster. Areas are presented by contrast and within a contrast sorted from anterior to posterior and from ventral to dorsal. Full activation maps for all areas shown in the table are available from the authors*.

Next, we were interested in examining *amygdala* activity to happy compared to neutral or angry faces across the whole sample (see Table 1; *Hypothesis 1b*). As show in Table 4 (section A, Analysis across whole sample), somewhat similar to the findings in vmPFC, we found significant right amygdala activity for *happy faces* > *neutral faces* (MNI: *x* = 24, *y* = −9, *z* = −12) and for *happy faces* > *angry faces* (MNI: *x* = 24, *y* = −9, *z* = −18). Follow-up tests collapsed across the whole sample on extracted beta values at the peak voxel of activation showed that right amygdala activity (MNI: *x* = 24, *y* = −9, *z* = −12) was greater for happy than neutral faces [*t*(61) = 2.97, *p* = 0.004]. Again, the pattern of results was comparable for the two age groups: young [*t*(29) = 2.26, *p* = 0.031] and, marginally, older [*t*(31) = 1.97, *p* = 0.058] participants showed greater amygdala activity for happy than neutral faces. Note that contrasting *young faces* > *older faces* resulted in no significant brain activity in any area of the examined ROIs (see *Hypotheses 1a* and *1b*).

##### Neutral faces > happy faces, angry faces > happy faces, and older faces > young faces across the whole sample

The next set of analyses addressed whether there was greater dmPFC activity to neutral or angry compared to happy faces and to older compared to young faces across the whole sample (see Table 1; *Hypothesis 1c*). As shown in Table 4 (section A, Analysis across whole sample), when contrasting neutral or angry with happy faces, a region of left *dmPFC* showed greater BOLD response. This dmPFC region was very similar for both neutral greater than happy faces (MNI: *x* = −6, *y* = 24, *z* = 48; note that for this contrast, the activity was bilateral) and angry greater than happy faces (MNI: *x* = −6, *y* = 15, *z* = 51). Figure 4A shows activity in dmPFC for the contrast *neutral faces* > *happy faces* (MNI: *x* = −6, *y* = 24, *z* = 48). Follow-up tests across the whole sample on extracted beta values at the peak voxel of activation showed that left dmPFC activity was greater for neutral [*t*(61) = 4.38, *p* < 0.001] and angry [*t*(61) = 5.85, *p* < 0.001] than happy faces. Figure 4B shows comparable results when examining young and older participants separately with both young [*t*(29) = 4.15, *p* < 0.001] and older [*t*(31) = 2.59, *p* = 0.014] participants showing greater dmPFC activity for neutral than happy faces.

In addition, for *older faces* > *young faces* bilateral *dmPFC* showed greater BOLD response to older than young faces (see Table 4, section A, Analysis across whole sample). This region of dmPFC (MNI: *x* = −3, *y* = 33, *z* = 39) was very similar to the dmPFC region (MNI: *x* = −6, *y* = 24, *z* = 48) reported above for *neutral faces* > *happy faces*. Figure 4D shows this activity in dmPFC (MNI: *x* = −3, *y* = 33, *z* = 39) for the contrast *older faces* > *young faces*. Follow-up paired-sample *t*-tests across the whole sample on extracted beta values at the peak voxel of activation showed that activity in left dmPFC was greater for older than young faces [*t*(61) = 4.60, *p* < 0.001]. Figure 4E presents the data separately for young and older adults and shows that for older [*t*(31) = 4.90, *p* < 0.001], but only marginally young [*t*(29) = 1.96, *p* = 0.060], participants left dmPFC activity was greater for older than young faces. Note that even at the lower threshold (*p* < 0.05 uncorrected, number of contiguous voxels unspecified), there was no significant amygdala activation for this set of contrasts.

##### Happy faces vs. neutral faces and happy faces vs. angry faces in interaction with participant age group

Based on previous research, for a next set of analyses we had the specific hypothesis that there would be greater dmPFC activity to neutral or angry than happy faces in older than young adults (see Table 1; *Hypothesis 1d*). We also examined participant age-group differences in vmPFC and amygdala activity, but we did not have specific hypotheses for these analyses given rather mixed previous literature (see, e.g., Leclerc and Kensinger, 2008; St Jacques et al., 2009). Table 4 (section B, Interaction with participant age) summarizes the areas of mPFC that showed age-group differences in activity to happy vs. neutral and/or happy vs. angry faces (*F*-contrasts). These analyses suggest that, even though we saw similar patterns of brain activity between young and older adults in the analyses reported above, there also were some age-group differences in the recruitment of subregions of vmPFC and dmPFC in the present task: In particular, as shown in Figure 5A, for the *F*-contrast *happy faces* vs. *angry faces by age of participant*, a region of vmPFC (MNI: *x* = 12, *y* = 39, *z* = −3), that was slightly more posterior and more lateral than the vmPFC regions presented in Figure 3A (MNI: *x* = −3, *y* = 57, *z* = −3), showed greater activity to happy than angry faces [*t*(29) = 2.99, *p* < 0.001] and also to neutral faces [*t*(29) = 3.18, *p* = 0.004] in young adults. For older adults, however, brain activity in this more posterior and lateral region of vmPFC did not differ between happy and angry [*t*(31) = −0.54, *p* = 0.593] nor happy and neutral [*t*(31) = 0.095, *p* = 0.925] faces (see Figure 5B). By contrast, as shown in Figure 5C, activity in an area of dmPFC (MNI: *x* = −6, *y* = 27, *z* = 51), that was largely overlapping with the dmPFC region presented in Figure 4A (MNI: *x* = −6, *y* = 24, *z* = 48), was greater in response to angry than happy [*t*(31) = −4.98, *p* < 0.001], as well as to neutral than happy [*t*(31) = −2.60, *p* = 0.014] faces in older participants. Note that activity in this area of dmPFC in young participants was only marginally greater in response to angry than happy faces [*t*(29) = −1.92, *p* = 0.065], but was significantly greater in response to angry than neutral faces [*t*(29) = −3.28, *p* = 0.003; see Figure 5D]. There was no significant amygdala activity for these contrasts, even when lowering the threshold (*p* < 0.05 uncorrected, number of contiguous voxels unspecified).

#### Brain-behavior correlations

With respect to the brain-behavior correlations, we were particularly interested in examining whether brain responses to one facial expression in relation to another were correlated with the ability to read one expression in relation to another. This approach required use of difference scores. That is, brain activity resulting from contrasting one facial expression with another (e.g., *happy faces* > *angry faces*) was correlated with behavioral performance (accuracy and speed, respectively) for one facial expression (e.g., happy) contrasted with another (e.g., angry).

First, we tested whether there were positive correlations between *vmPFC* activity to happy relative to neutral or angry faces and accuracy and speed, respectively, of identifying happy relative to neutral or angry faces across the whole sample as well as for young and older adults separately. We tested the same pattern of findings for young vs. older faces (see Table 1; *Hypothesis 2a*). As expected, the difference in BOLD response to happy vs. angry faces in the observed area of left vmPFC (MNI: *x* = −3, *y* = 57, *z* = −3) was positively correlated with the difference in accuracy in reading facial emotions of happy relative to angry faces across participants (Pearson *r* = 0.36, *p* = 0.005). As shown Figure 3C, examining young and older participants separately, this positive correlation was significant in young (Pearson *r* = 0.47, *p* = 0.010), but only marginally in older (Pearson *r* = 0.31, *p* = 0.092), participants. In addition, the greater the BOLD response to happy relative to angry faces in this region of left vmPFC, the faster young participants (response time: Pearson *r* = −0.46, *p* = 0.011) were able to read happy relative to angry facial expressions. This correlation was not significant in the older participants and was not significant when collapsing across the whole sample.

Next, we examined whether there were positive correlations between *amygdala* activity to happy relative to neutral or angry faces and accuracy and speed of identifying happy relative to neutral or angry faces across the whole sample and for young and older adults separately. Again, we tested the same pattern of findings for young vs. older faces (see Table 1; *Hypothesis 2b*). BOLD response to happy relative to angry faces in right amygdala (MNI: *x* = 24, *y* = −9, *z* = −18) was positively correlated with participants’ accuracy (Pearson *r* = 0.35, *p* = 0.006) in reading facial expressions of, and the faster they were in responding to (response time: Pearson *r* = −0.25, *p* = 0.05), happy compared to angry faces. Investigating young and older participants separately, we found positive correlations for older (Pearson *r* = 0.39, *p* = 0.03), but only marginally for young (Pearson *r* = 0.31, *p* = 0.10), participants in their accuracy in reading facial expressions of happiness relative to anger, but no significant correlations with speed of responding.

Finally, we examined whether there were negative correlation between *dmPFC* activity to neutral or angry faces relative to happy faces and accuracy and speed of identifying neutral or angry faces relative to happy faces across the whole sample, as well as for young and older adults separately. The same pattern of findings was tested for older relative to young faces (see Table 1; *Hypothesis 2c*). The difference in BOLD response to neutral relative to happy faces in left dmPFC (MNI: *x* = −6, *y* = 24, *z* = 48) was negatively correlated with participants’ accuracy in reading neutral relative to happy facial expressions (Pearson *r* = −0.38, *p* = 0.008), and the greater the brain activity in left dmPFC, the slower were participants in giving their responses (response time: Pearson *r* = 0.41, *p* = 0.002). As shown in Figure 4C, examining young and older participants separately, this difference in BOLD response to neutral relative to happy faces in left dmPFC was negatively correlated with older (Pearson *r* = −0.36, *p* = 0.049), but only marginally with young (Pearson *r* = −0.32, *p* = 0.086), participants’ accuracy in reading neutral compared to happy facial expressions. In addition, the greater the BOLD response to neutral relative to happy faces in this region of left dmPFC, the slower older (response time: Pearson *r* = 0.51, *p* = 0.004) but not young participants read neutral relative to happy expressions. Note that we found no significant correlations with BOLD response to *young faces* > *older faces* or *older faces* > *young faces* in any of the examined regions and behavioral performance, neither across young and older participants, nor for the age groups separately (see *Hypotheses 2a*–*2c*).

## Discussion

The central goal of the present study was to increase knowledge of the neural mechanisms underlying identification of positive, neutral, and negative expressions in young and older adult faces. In particular, we were interested in investigating samples of young and older adults with respect to the neural correlates of reading facial emotions. The study examined the role of mPFC and amygdala, brain areas associated with facial emotion processing, while young and older adults engaged in facial expression identification. Targeting the functional role of these selected brain regions, the study directly examined the correlations between activity in mPFC as well as amygdala to specific facial expressions relative to others, and young and older adults’ ability to correctly read one expression over the other. The present study provides converging evidence for previous observations and reports several novel findings.

### Young and older adults were better and faster at reading emotions from happy and young faces

Young and older adults in the present study did not differ in their accuracy of reading facial emotions. This finding differs somewhat from previous studies (see Ruffman et al., 2008, for an overview). However, in contrast to previous studies, the present study only used three different facial expressions (i.e., happy, neutral, angry). This was done to increase comparability in stimulus and response variety for positive and negative expressions, that is, to better equate task complexity for positive and negative expressions (as compared to using one positive along with various negative expressions, which likely results in qualitatively different tasks for identification of positive and negative expressions; see Ebner et al., 2011c). Thus, overall, the present task was likely easier than paradigms used in previous work, as reflected in the high accuracy and fast response under all conditions for both young and older adults in the present study (see Figure 2 and Table 3).

Importantly, even though there were no age-group differences in accuracy, in line with age-related decline in the ability to read facial expressions, older compared to young adults were slower in responding to happy and angry, but not neutral, faces. Moreover, consistent with prior studies (Ebner and Johnson, 2009; Ebner et al., 2011c; Riediger et al., 2011), both young and older adults were more accurate and faster in reading happy than neutral or angry faces. Both age groups were also more accurate and faster in reading expressions in young than older faces. That is, in line with previous work (Ebner and Johnson, 2009; Ebner et al., 2011c), young adults were better at reading expression of faces of their own-age group, but for older adults’, there was no indication of an own-age advantage in facial emotion reading. Similar findings have been explained in terms of greater complexity and more ambiguity of neutral and angry compared to happy (Ebner and Johnson, 2009) and older compared to young (Ebner et al., 2011b) faces. Slower response time to angry than neutral faces for older but not young participants (and only for young but not older faces; see significant three-way interaction in Figure 2C) suggests that for older adults, it may be particularly hard differentiating neutral from angry young but not older faces, maybe because anger is an expression that an older person would not expect to see or is reluctant to attribute to a young person’s face.

Taken together, in line with the literature, the present study provides supporting evidence of both young and older adults’ greater ability to read emotions from happy compared to neutral or angry and from young compared to older faces.

### Ventral/dorsal distinction in mPFC during reading facial emotions in young and older adults and brain-behavior correlations

Importantly, the behavioral differences in the ability to read expressions of happy, neutral, and angry young and older adult faces were reflected in young and older adults’ neural responses. In particular, there was greater *vmPFC* activity in response to happy than neutral or angry faces in both young and older participants. In addition, this greater vmPFC activity to happy compared to angry faces was positively correlated with the ability to read happy relative to angry faces. Specifically, greater vmPFC activity to happy relative to angry faces was positively correlated with accuracy of reading facial happiness opposed to facial anger in both young and older participants. In addition, the greater the vmPFC activity to happy relative to angry faces, the faster young participants were able to read happy compared to angry faces. These findings are consistent with the idea that happy expressions, compared to other expressions, are readily available and easy to process. Salience may derive, in part, from a reward value associated with happy faces. That is, it is possible that activity in this area of vmPFC reflects *affective response*, and in particular, positive affective response, to “good” cues, such as the happy compared to the neutral or angry faces in the present task. Support for this interpretation comes from Mitchell et al. (2009), who found greater activity in a very close area of vmPFC (MNI: *x* = 0, *y* = 52, *z* = −11) during self-relevant thought in both young and older adults, and in particular, showed greater activity in this region of vmPFC when young adults thought about more positive compared to negative personal agendas. It is also supported by Kim and Johnson (2012), who found greater activity in a largely overlapping subregion of vmPFC (MNI: *x* = 2, *y* = 52, *z* = −4) when young participants were randomly assigned objects compared to when objects were assigned to another person, and, importantly, found an association of this vmPFC activity with increased preference for objects assigned to the self.

Similar to the findings for vmPFC, there was greater *amygdala* activity to happy than neutral or angry faces in both age groups. Also, amygdala activity to happy compared to angry faces was positively related to accuracy in, and faster response during, reading of happy relative to angry faces. This finding may appear somewhat counter-intuitive in light of findings that angry (and fearful) faces typically activate amygdala more than neutral or happy faces (Whalen et al., 2001), and that amygdala has been discussed as involved in processing ambiguity in faces (Davis and Whalen, 2001). However, our finding is in line with results by Keightley et al. (2007) and other studies that provide evidence that amygdala is also responsive to positive faces (Hamann et al., 2002; Yang et al., 2003; Zald, 2003). Thus, the direction of our amygdala finding further supports the notion of greater positive affective response to happy compared to neutral or angry faces. It is in accord with evidence of greater amygdala response to faces that are associated with more positive evaluations, greater familiarity, and more self-relevance (see Van Bavel et al., 2008; Wright et al., 2008; Ebner et al., in preparation), like the happy compared to the neutral or angry faces in the present context. Thus, this finding lends further support to a role of amygdala, and possibly in connection with vmPFC, in a wider range of emotional processing than simply processing of negative information (see also Shaw et al., 2005; Keightley et al., 2007). Our findings of greater amygdala response to happy than angry (or neutral) faces, instead of greater amygdala activity to negative than positive stimuli (Whalen et al., 2001), is furthermore in line with D:Lieberman et al. (2007) finding of diminished amygdala activity during labeling compared to passively viewing of emotional faces in a sample of young adults. In line with Lieberman et al.’s interpretation, it is possible that in our study, the more cognitively demanding process of identifying angry and neutral than happy expressions dampened amygdala response. This process may be modulated by mPFC, and in particular, dmPFC as discussed below (cf. Lieberman et al., 2007).

By contrast, comparison of brain activity to neutral or angry with that associated with happy faces resulted in greater *dmPFC* activity for both age groups. Exploring again the brain-behavior correlations, we found that greater dmPFC activity to neutral than happy faces was associated with less accurate and slower expression identification for neutral relative to happy faces. Importantly, a very similar region of dmPFC also showed greater activity for older than young faces, with no correlations between brain activity and behavioral performance.

Taken together, the pattern of findings observed in the present study suggests an important functional dissociation between vmPFC, possibly in interaction with amygdala, and dmPFC in facial emotion reading. And importantly, this functional dissociation is quite comparable between young and older adults. There is evidence that vmPFC is associated with affective and valenced evaluative processing (Bush et al., 2000; Cunningham et al., 2004; Ochsner et al., 2005; Lebreton et al., 2009; Kim and Johnson, 2012). In contrast, there is evidence that dmPFC is recruited during more cognitively complex processing (see also Amodio and Frith, 2006; Northoff et al., 2006; Van Overwalle, 2009). In particular, dmPFC and dorsal anterior cingulate have been found to be involved in a variety of tasks requiring cognitive control (Bush et al., 2000; Carter et al., 2001; Paus, 2001). Thus, increased activity in dmPFC to neutral and angry compared to happy faces likely reflects *increased cognitive control* to identify (and perhaps differentiate between) angry and neutral expressions. It is possible that this differential dmPFC activity in response to happy vs. angry or neutral faces directly interacts with vmPFC and amygdala response to these stimuli. In particular, the greater mental effort of identifying angry or neutral relative to happy faces, which is associated with greater dmPFC activity, may result in decreased affective response (reflected in decreased vmPFC and amygdala activity) to angry or neutral compared to happy faces.

Very interesting in the context of the present study was also the highly overlapping pattern of brain activation for angry/neutral relative to happy faces and older relative to young faces, respectively, for both young and older participants. This is particularly intriguing as angry/neutral and older faces were the faces that were harder to read for both young and older adults. Thus, this further supports that the ventral/dorsal mPFC dissociation seen in the present study (and similarly in Keightley et al., 2007) reflect differences in demands for cognitive control, perhaps due to differences in the availability of facial cues necessary for accurate expression identification in happy compared to neutral or angry faces.

Thus, overall, the observed ventral/dorsal distinction in mPFC was quite comparable in young and older adults. However, at the same time, we also saw some informative differences in young and older adults’ brain response during the present study’s facial expression identification task: In particular, young but not older adults showed greater activity in a more posterior, more lateral subregion of vmPFC (Figure 5A) in response to happy compared to angry faces. This suggests that there may be a functional difference between this more posterior, more lateral vmPFC region and the more anterior and less lateral vmPFC region in which young and older adults showed largely the same pattern (Figure 3A). Also, it is possible that in older compared to young adults, a less extensive subregion of vmPFC (the more anterior, more medial part of vmPFC) is involved in differentiating between happy, neutral, and angry expressions.

Importantly, we also found further evidence that increased activity in dmPFC to angry (and neutral) compared to happy faces was more pronounced in older than young adults. This age-group difference is in accord with our hypothesis that greater dmPFC activity to angry than happy faces in older compared to young adults may reflect older adults’ particular difficulty reading angry faces (Ebner and Johnson, 2009; Ebner et al., 2011c; see also Ruffman et al., 2008), as identification of angry faces requires more complex judgment. Alternatively, this finding is in line with evidence of an age-associated increase in controlled regulatory processing of negative than positive faces (Williams et al., 2006), a finding consistent with an increased positivity bias in older compared to young adults as suggested by *Socio-emotional Selectivity Theory* (Carstensen et al., 1999; Carstensen, 2006).

Our observed age-group differences seem at odds with results reported by Gutchess et al. (2007) and by Leclerc and Kensinger (2008). Gutchess et al. found greater dmPFC activity in older (but not young) adults during processing of (self-referential) positive than negative information. Leclerc and Kensinger, in line with our findings, found greater vmPFC activity during processing of positive than negative information in older adults, but found greater vmPFC activity during processing of negative than positive information in young adults. Also, previous studies observed a subcortical to cortical shift with age during emotion processing (Iidaka et al., 2001; Gunning-Dixon et al., 2003; Fischer et al., 2005; Tessitore et al., 2005), whereas the present study found increased amygdala, in addition to increased vmPFC, activity to happy compared to neutral or angry faces for both young and older adults. When comparing these studies, it is important to consider that they differed substantially in design (e.g., block- vs. event-related design, orienting tasks and stimulus material used). Gutchess et al. for instance, used positive vs. negative person-descriptive adjectives in the context of a self- and other-evaluation task, and Leclerc and Kensinger used images of positive and negative objects in the context of an object categorization task not related to valence.

The studies by Keightley et al. (2007) and Williams et al. (2006) are most similar in approach to the present study and they produced quite comparable results. At the same time, there also were some design-related differences between their studies and ours that may explain some differences in the findings. For instance, different from Keightley et al. and Williams et al., the present study focused on happy, neutral, and angry facial expressions. Limiting the investigation to only these three facial expressions resulted in sufficient trials to allow direct comparison of each expression. Also, focusing on only three expressions, and the fact that we used an event-related design not a block-design as in Williams et al., made corresponding button presses feasible in the scanner. This allowed us to directly link brain activity during task engagement to behavioral responses, instead of having to refer to outside-the-scanner (re-)labeling of the facial expressions. Also, faces did not have to be presented twice (prior/post and during scanning), which may have induced participants to try to remember which expression they had assigned to a face when initially presented with it. Furthermore, in the present study, the identical face identities were presented in all three emotion expressions, counterbalanced across participants. This increased the control over varying levels of complexity of the face stimuli as a function of the different emotions displayed. The present study examined a larger sample of young and older adults than the other two studies, contributing to the reliability of the findings. And, importantly, whereas all previous fMRI studies on age-group differences in facial expression identification exclusively used young and no older faces, the present study systematically varied the age of the presented faces and thus could explicitly address age-of-face effects.

### Conclusion and outlook

The specific focus of the present study was to examine the neural mechanisms involved in reading emotions. Extending previous work, we examined neural correlates of facial expression identification in two populations (young and older adults) and with respect to faces that differed in valence (happy, neutral, and angry faces) as well as age (young and older faces). Thus, we were able to examine the comparability as well as the differences in the neural underpinnings of facial emotion reading between young and older adults as a function of the facial expression and the age of the faces, respectively. In addition, our design allowed us to directly examine correlations between brain activity and behavioral task performance.

The present study adds to the knowledge about the neural mechanisms of facial expression identification in young and older adults and extends earlier work in several important ways. It provides important new evidence of a *ventral/dorsal distinction in mPFC, possibly in interaction with amygdala, during facial emotion reading in both young and older adults*: Increased vmPFC and amygdala activity may reflect increased affective processing, and maybe positive affective processing in particular, of the more salient, unambiguous, and positive happy faces. In contrast, increased dmPFC activity may reflect increased cognitive effort to decode the more ambiguous and complex neutral or angry as well as older faces and/or to regulate negative emotions associated with such faces, particularly in older adults. And it is possible that this increased effort in identifying neutral and angry compared to happy faces results in dampened response in vmPFC and amygdala. The interpretation of affective vs. cognitive processing in vmPFC vs. dmPFC, respectively, was further supported by brain-behavior correlations: There was a positive correlation between vmPFC activity to happy relative to angry faces and a negative correlation between dmPFC activity to neutral relative to happy faces and the ability to read the respective facial expression over the other, with largely comparable correlations in young and older adults.

There are various promising routes to take this work in the future: One such future direction is to examine age-related changes in brain structure, such as reductions in gray volume and/or white matter integrity in regions of the brain associated with facial emotion reading, and their correlations with functional brain changes and behavioral performance. This approach may be particularly interesting given evidence that the vmPFC and amygdala only undergo relatively modest structural changes with age (Shimamura, 1994; West, 1996; Hedden and Gabrieli, 2004). Moreover, there are suggestions that vmPFC vs. dmPFC show somewhat different rates of structural and functional age-related decline, with dmPFC (like lateral PFC regions) exhibiting earlier and somewhat more rapid decline in normal aging than other regions of PFC (Daigneault and Braun, 1993; Shimamura, 1994; West, 1996). The empirical work on structure-function relations in the context of facial expression identification in young and older adults is, to date, still very limited. Williams et al. (2006) found support for age-related gray matter volume declines in mPFC, while there was comparative preservation of the amygdala and the basal ganglia caudate region. However, even though the age-related loss of mPFC gray matter predicted decline in correct post-scan identification of fearful faces, no correlation between structural and functional brain changes were observed. Another important future avenue is to address the predictive value of brain activity during, and performance in, facial emotion reading assessed in the laboratory context for successful real-life social interactions, social embeddedness, and socio-emotional well-being. Investigation of these effects may be particularly interesting in adults of different ages as well as in clinical populations that experience particular difficulties with reading facial expressions (e.g., autism) or that have a particular bias toward certain emotions (e.g., depression). Finally, a better understanding of the mechanisms involved in correct interpretation vs. misinterpretation of emotional facial expressions in others, and how certain confusions of facial emotions may differently influence (hinder or promote) attention to, and memory for, faces in young and older adults (see Ebner and Johnson, 2009; Ebner et al., 2011c) is warranted in the future.

## Conflict of Interest Statement

The authors declare that the research was conducted in the absence of any commercial or financial relationships that could be construed as a potential conflict of interest.

## Footnotes

^1^http://www.nitrc.org/projects/wfu_pickatlas/

^2^http://www.brainmap.org/icbm2tal/
